# Safety of selective nonoperative management for blunt splenic trauma: the impact of concomitant injuries

**DOI:** 10.1186/s13037-018-0179-8

**Published:** 2018-11-27

**Authors:** Michel Paul Johan Teuben, Roy Spijkerman, Taco Johan Blokhuis, Roman Pfeifer, Henrik Teuber, Hans-Christoph Pape, Luke Petrus Hendrikus Leenen

**Affiliations:** 1 Department of Trauma, University Medical Centre Utrecht, Heidelberglaan 100, 3584 CX Utrecht, The Netherlands; 2 Department of Surgery, Maastricht University Medical Center, P. Debyelaan 24, 6229 HX Maastricht, The Netherlands; 3 Department of Trauma, University Hospital Zurich, Raemistrasse 100, 8091 Zürich, Switzerland

**Keywords:** Blunt splenic injury, Abdominal trauma, Nonoperative management, Concurrent injuries

## Abstract

**Background:**

Nonoperative management for blunt splenic injury is the preferred treatment. To improve the outcome of selective nonoperative therapy, the current challenge is to identify factors that predict failure. Little is known about the impact of concomitant injury on outcome. Our study has two goals. First, to determine whether concomitant injury affects the safety of selective nonoperative treatment. Secondly we aimed to identify factors that can predict failure.

**Methods:**

From our prospective trauma registry we selected all nonoperatively treated adult patients with blunt splenic trauma admitted between 01.01.2000 and 12.21.2013. All concurrent injuries with an AIS ≥ 2 were scored. We grouped and compared patients sustaining solitary splenic injuries and patients with concomitant injuries. To identify specific factors that predict failure we used a multivariable regression analysis.

**Results:**

A total of 79 patients were included. Failure of nonoperative therapy (*n* = 11) and complications only occurred in patients sustaining concomitant injury. Furthermore, ICU-stay as well as hospitalization time were significantly prolonged in the presence of associated injury (4 versus 13 days,*p* < 0.05). Mortality was not seen. Multivariable analysis revealed the presence of a femur fracture and higher age as predictors of failure.

**Conclusions:**

Nonoperative management for hemodynamically normal patients with blunt splenic injury is feasible and safe, even in the presence of concurrent (non-hollow organ) injuries or a contrast blush on CT. However, associated injuries are related to prolonged intensive care unit- and hospital stay, complications, and failure of nonoperative management. Specifically, higher age and the presence of a femur fracture are predictors of failure.

## Background

The spleen is the most frequently injured organ in blunt abdominal trauma [1, 2]. In the past, splenic injuries were routinely treated by splenectomy. Increasing awareness of the spleen’s role in the immune system and the recognition of overwhelming sepsis after splenectomy (overwhelming post splenectomy infection) resulted in an upsurge of interest in the preservation of splenic function [3–5]. In the last two decades, nonoperative management of blunt splenic injuries in hemodynamically stable patients has become the preferred treatment [5–7].

The shift towards nonoperative treatment was initiated by Upadhyaya and Simpson. They were the first to report successful observational management in children in 1968 [8]. Additional favorable results from pediatric series followed and the strategy was gradually introduced in adult surgery. Nowadays, selective nonsurgical treatment of splenic injury can be expected to be successful in over 90% of cases [5, 9–13].

The most important prerequisite for successful selective nonoperative management is adequate patient selection. Therefore, the current challenge is to identify factors that predict failure. A considerable amount of literature has been published on this subject. The key criteria to select patients for nonoperative management is hemodynamic stability.

However, higher age [12, 14–16], the presence of vascular blush on Computed Tomography (CT) scanning [17, 18], higher Splenic Injury Grade [19, 20], large quantity of hemoperitoneum [20, 21] and higher Injury Severity Score (ISS) have been identified as factors associated with failure of nonoperative management [14, 21–23] as well. Due to conflicting results these factors are still subject to debate.

According to the novel World Society of Emergency Surgery (WSES) guidelines, the optimal treatment strategy in patients with splenic injury should take hemodynamic status, anatomic derangement and concomitant injuries into consideration [6].

To date, little is known about the impact of concomitant injury on outcome. For a long time polytrauma was a relative contraindication for nonoperative treatment. Although, more recent series conclude that nonoperative therapy may be attempted in adult patients sustaining multiple injuries without increased morbidity [24, 25].

In our level one trauma center, all hemodynamically stable patients without signs of hollow viscus injury are considered candidates for nonoperative treatment, regardless of splenic injury grade, polytrauma (higher Injury Severity Score), the presence of severe associated injuries (such as craniocerebral or thoracic), or higher age of the injured patient. Due to ambiguous results in the current literature, our study had two objectives. The first objective was to determine whether associated injury affects the safety of nonoperative management for blunt splenic trauma. Secondly, we tried to identify specific factors and/or associated injuries that can predict failure.

## Methods

From a prospective trauma database we selected all patients (≥16 years) with blunt splenic injury presenting to our level one trauma center between January 1, 2000 and December 21, 2013. In our institution all patients aged ≥16 years are treated according to the same standardized resuscitation and shock room guidelines. These guidelines did not change during the study period. In addition, all patients were treated by the same group of specialized trauma physicians. As 16- and 17-year-old patients are allowed to have full time jobs and to have licenses to drive motorized vehicle,s they are at risk for comparable (high-enegery) trauma mechanisms as older patients. Therefore these patients are treated according to treatment standards for adults and included in our study.

**Fig. 1:**
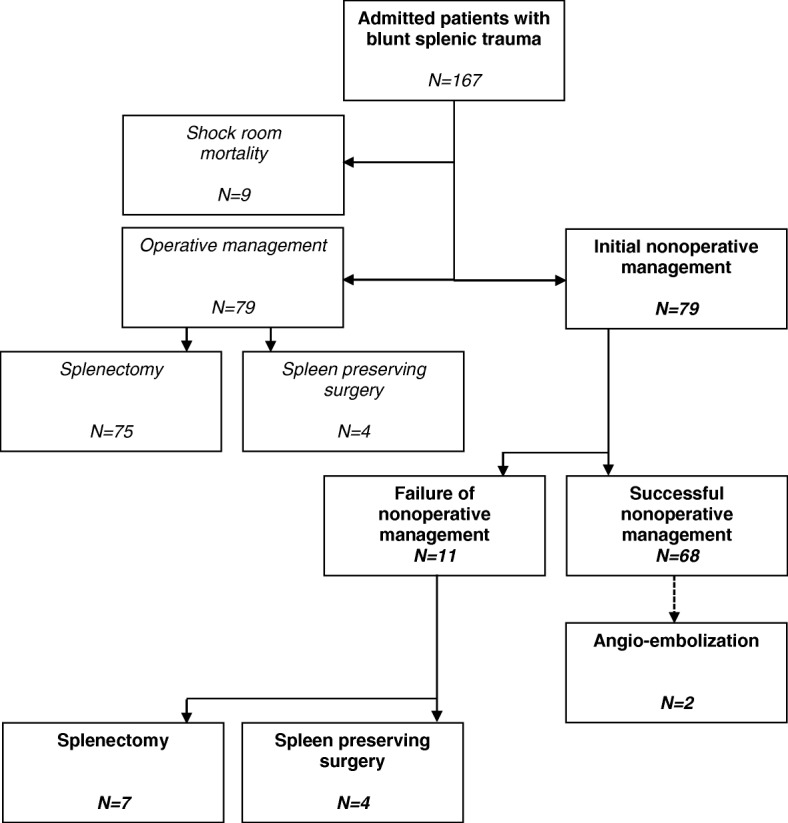
Flowchart

Patients who required immediate emergency laparotomy or died before total diagnostic workup was completed, were excluded from the study. A flow diagram summarizes all inclusions and exclusions from the primary trauma cohort (Fig. 1). After providing initial resuscitation and management based upon protocols from Advanced Trauma Life Support*,* patients underwent CT-scanning (or when the CT-scan was not available an ultrasound investigation).

In our trauma centre, all hemodynamically normal patients without signs of hollow viscus injury were selected for nonoperative management, regardless of splenic injury grade, the presence of a contrast blush on CT, polytrauma conditions, the presence of severe associated injuries, or age of the patient. Those individuals selected for nonoperative management underwent CT scanning (or an ultrasound investigation in the case CT-scanning was not available).

Failure of nonoperative management was defined as any situation in which a patient who was initially admitted to the Intensive Care Unit or general ward for nonoperative management later required laparotomy.

Patient demographics, mechanism of injury, Injury Severity Score, Glasgow Coma Scale (GCS), hemodynamic parameters at arrival, concomitant injury, management and outcome were reviewed.

To analyze the impact of early coagulopathy on outcome, we used the criteria described by Macleod et al. [26]. Early coagulopathy was defined as the presence of prothrombin time (PT) > 14 s or Activated Partial Thromboplastin Time (APTT) > 34 s.

Injuries were described based on radiographic imaging or operative findings and according to the Abbreviated Injury Score dictionary, or the WSES-classification [6, 27]. We identified solid organ intra-abdominal (kidney, liver, pancreas), hollow organ intra-abdominal (stomach, small bowel, colon), thoracic, spinal, femoral bone, pelvic, maxillofacial and craniocerebral injuries. All injuries with an abbreviated injury score of at least two were documented.

Solitary rib injuries were excluded, because they are not believed to be sources of major hemorrhage, or to influence management and outcome directly.

To determine the influence of associated injury on outcome we grouped and compared patients sustaining solitary splenic injury (group I) and patients with significant concomitant injury (group II). Concurrent injuries were considered as significant if they scored an abbreviated injury score of 2 or more.

We compared the length of intensive care unit stay, total hospital length of stay, failure of nonoperative treatment, complications, and mortality.

To identify which factors predicted failure we used a backward stepwise logistic regression analysis. First, univariable analysis was performed and all factors with a positive *p*-value of less than 0.2 were selected for multivariable analysis. A backward stepwise logit regression analysis was performed and our model was validated by a forward regression analysis in which comparable results were found.

For continuous data, the median and 25th to 75th inter quartile range (IQR) or range, were reported. Categorical data are presented in frequencies, unless otherwise stated. Normally distributed continuous data were analyzed with the Student’s unpaired sample t-test. Non-normally distributed data were compared by using the parametric-free Mann-Whitney U test and the Pearson Chi-square (dichotomized data). The Yates’ continuity correction was applied to correct for scrutiny. *P*-values 0.05 or less were regarded as statistically significant. All computations were performed using the Statistical Package for Social Sciences software for Windows (SPSS for Windows 20.0).

## Results

During the study period a total of 79 (64 men and 15 women) hemodynamically stable patients sustaining blunt splenic injury were selected for nonoperative management. Patient age ranged from 16 to 81 years, and the median (IQR) age was 28 (20–53).

The mechanisms of injury included 21 motorcycle accidents, 20 motor vehicle accidents, 14 bicycle accidents, 8 falls from a height, 2 pedestrians hit by a vehicle, and 14 miscellaneous (including sports and assault injuries). The median Glasgow Coma Scale score was 15. A grade I-III splenic injury was present in 65 patients and 14 patients had a grade IV-V splenic injury (Table 1). Admission median (IQR) systolic blood pressure was 130 (120–140), median mean arterial pressure (IQR) was 95 (86–102) and the median (IQR) heart rate was 88 (76–100). Median (IQR) serum hemoglobin was 8.1 (7.1–8.6) and median (IQR) hematocrit of the patient population was 0.38 (0.34–0.41).

**Table 1:** Patient characteristics

Variable	*n = 79*
Age in years (IQR)	28 (19–51)
Gender ratio (M/F)	64/15
Systolic blood pressure in mmHg (IQR)	130 (120–140)
Heart rate in BPM (IQR)	88 (76–100)
Glasgow coma score (range)	15 (3–15)
Injury Severity Score (IQR)	18 (9–79)
Spleen injury scale	
I-II	45
III	20
IV	11
V	3

All data; Median (IQR/range). *BPM* beats per minute

A total of 66 patients suffered concomitant injury, of whom 40 had more than one associated injury. Concurrent intra-abdominal injuries were found in 30 patients and extra-abdominal injuries were present in 53 patients. The most frequent concomitant injury was thoracic injury (34 out of 79 patients). Injuries of other solid intra-abdominal organs occurred frequently as well. Twenty-one patients had concomitant kidney injuries (16 left and 5 right) and 16 hepatic injuries occurred. Eight patients sustained both kidney and liver injuries in addition to their splenic injury. Nine patients had associated spinal injuries, of whom one patient had both lumbar and thoracic spinal injuries. Five patients had injuries of the lumbar spine, and both the thoracic spine and cervical spine were injured in two patients each. Table 2 shows all associated injuries that were found.

**Table 2:** Frequency of associated injuries

Associated injuries	Frequency
*Intra abdominal*	
Kidney left	16
Liver	16
Kidney right	5
Mesentery	2
*Extra abdominal*	
Thorax	34
Craniocerebral	15
Pelvic bone	13
Spine	9
Maxillofacial	8
Femur bone	3

### Impact of concomitant injury on outcome

Demographics and outcome between patients sustaining concurrent injuries, and those patients without associated injuries are summarized in Table 3. As anticipated, there was a significant difference in Injury Severity Score between the solitary and associated injury groups. No statistically significant differences were found in age, gender distribution, admission systolic blood pressure, admission heart rate, the presence of a contrast blush on CT imaging, or grade of splenic injury between groups.

**Table 3:** Patient demographics, admission hemodynamic parameters, GCS, Injury severity and outcome in patients with solitary splenic injury (group I) compared with patients sustaining associated injuries (group II)

Variable	Group I: solitary splenic injury (*n* = 13)	Group II: associated injury (*n* = 66)
Age in years (IQR)	26 (18–59)	28 (20–50)
Gender ratio in M/F	8/5	56/10
Systolic BP in mmHg (IQR)	136 (111–153)	130 (120–140)
Heart rate in BPM (IQR)	88 (65–90)	89 (76–101)
Serum haemoglobin in mmol/L	8.3 (7.1–8.7)	8.4 (7.5–8.9)
Glasgow coma scale (range)	15 (7–15)	15 (3–15)
Injury Severity Score (IQR)^*^	4 (4–9)	20 (13–29)
Presence of a contrast blush on CT	0	5
AIS spleen (IQR)	2 (2–3)	2 (2–3)
Length of ICU stay in days (IQR)^*^	0 (0–3)	0.5 (0–6)
Hospitalization time in days (IQR)^*^	4 (4–8)	13 (9–27)
Failure of nonoperative treatment	0	11
Uncomplicated course	13/13	47/66
Total no. of major complications^*^	0	31
Mortality	0	0

All data; median (IQR) or (range), ^*^ *p* < 0.05, Mann-Whitney U test

Abbreviations: *AIS* abbreviated injury score, *BP* blood pressure, *ICU* intensive care unit

Failure of nonoperative therapy (*n* = 11) only occurred in patients suffering at least one concomitant injury. The development of hemodynamic instability resulted in failure of nonoperative management in eight patients. In three patients, a drop in serum hemoglobin combined with progressive hemoperitoneum resulted in the need for surgical intervention.

In five patients, failure occurred within 48 h of admission. In another four patients, failure occurred after 48 h of admission, and one failure occurred at day twelve. One individual with failure of nonoperative therapy at day three had a low grade splenic injury (Abbreviated Injury Score grade II/WSES class I). This patient did not undergo a CT-scan on admission and his splenic injury was therefore diagnosed at day three, after he had developed hemodynamic instability. Another patient required surgical intervention at day 5 due to a sudden drop in serum hemoglobin and presence of a contrast blush on computed tomography. This patient (WSES class II) was initially diagnosed with a grade III splenic injury and the initial CT-scan did not show active bleeding. Due to the rapidly deteriorating situation, both patients were treated with total splenectomy.

Two other patients (WSES class I and WSES class II) initially selected for nonoperative treatment with splenic injuries grades II and III respectively, failed nonoperative therapy after 48 h due to a severe drop in blood pressure. Both patients were treated with a spleen preserving procedure.

The patient who failed nonoperative management twelve days after trauma, presented to the hospital five days after initial trauma. He was previously diagnosed with chronic lymphocytic leukemia and sonography revealed a retroperitoneal hematoma. After twelve days of hospitalization, the patient developed hemodynamic instability and required emergency laparotomy in which a splenic hematoma was evacuated and a splenectomy was performed. Thereafter he required three further laparotomies for the treatment of intra-abdominal abscesses, and a further episode of hemodynamic instability due to a lesion of the pancreas. Those patients who failed nonoperative management were treated by a total of seven splenectomies and four spleen preserving procedures.

Complications were only encountered in patients sustaining associated injuries. The most frequent complications were pneumonia (*n* = 8) and ileus (*n* = 4). Two out of four patients developed ileus after splenectomy. In four patients, an intra-abdominal abscess was found and one of those abscesses was located in the perisplenic area of a nonoperatively treated patient.

In one patient, an intra-abdominal abscess was found near the pancreas after splenectomy. This was treated by CT-guided drainage two weeks after splenectomy (Table 4).

**Table 4:** Overview of complications

Complication	*n=*
Pneumonia	8
Ileus	4
Intra abdominal abscess	4
Respiratory failure	3
Acute Respiratory Distress Syndrome	3
Extra abdominal abscess	2
Line infection	2
Sepsis of unknown origin	1
Wound infection	1
Abdominal compartment syndrome	1
Urinary tract infection	1
Pulmonary embolus	1
Total	31

Duration of intensive care unit stay and hospitalization time were significantly prolonged in the presence of associated injury. The median length of hospital stay among the group with solitary splenic injury was 4 days, versus 13 days (*p* < 0.05) for patients sustaining concomitant injury. In both groups no mortality occurred.

### Factors associated with failure of nonoperative management

Univariable analysis of patient and trauma characteristics demonstrated age, Abbreviated Injury Score-spleen, and Injury Severity Score as relevant parameters (Table 5).

**Table 5:** Univariate analysis of patient and trauma characteristics

Variable	Successful nonoperative therapy *(n* = 68)	Failure of nonoperative therapy (*n* = 11)	*P*-value
Age in years*	*28 (19–50)*	*45 (22–60)*	*0.18*
SBP in mmHg	130 (120–140)	130 (15–145)	0.28
Heart rate in BPM	88 (76–99)	95 (75–110)	0.43
Serum hemoglobin in mmol/L	8.4 (7.5–8.9)	8.0 (7.0–8.4)	0.91
Coagulopathy	8/68	3/11	0.36
Glasgow coma scale	15 (14–15)	14 (9–15)	0.49
AIS-spleen*	*2 (2–3)*	*3 (2–4)*	*0.065*
Presence of a contrast blush	5/68	0/11	0.78
Injury Severity Score*	*17 (9–25)*	*32 (17–34)*	*0.003*

All data; median (IQR). ^*^ *p* < 0.2 Student’s t-test or Mann Whitney U test and selected for multivariable analysis

Abbreviations: *NOM* nonoperative management, *SBP* systolic blood pressure, *BPM* beats per minute, *AIS* Abbreviated Injury Score

Further analysis of concurrent injury showed that the presence of a femur fracture was also associated with failure (Table 6). Therefore, age, Injury Severity Score, Abbreviated Injury Score of splenic injury and the presence of a femur fracture were selected for multivariable analysis.

**Table 6:** Univariate analysis of concomitant injuries

Concomitant injury	Odds ratio	Lower (95% C.I.)	Upper (95% C.I.)
Abdominal solid organ	0.569	0.139	2.339
Craniocerebral	2.204	0.565	8.604
*Femur bone* ^a^	*14.889*	*1.223*	*181.251*
Maxillofacial	1.457	0.270	7.856
Pelvic bone	0.467	0.054	3.999
Spine	2.418	0.615	9.506
Thorax	2.657	0.709	9.958

^a^Selected for multivariate analysis

A stepwise backward logistic regression analysis revealed higher age as well as the presence of a femur fracture as independent predictors of failure. For each year increase in age, the chances of failure increases by a factor of 1.06. The presence of a femur fracture increased the odds of failure by a factor 25.9 (Table 7). Higher Abbreviated Injury Scores of diagnosed splenic injury, higher Injury Severity Scores were not significantly predictive of nonoperative treatment failures in our multivariable prediction model.

**Table 7:** Results of stepwise logit regression analysis for the risk of failure of nonoperative management

Variable	Odds ratio	Lower (95% C.I.)	Upper (95% C.I.)	*P*-value
Age	1.063	1.010	1.119	0.020
Femur fracture	25.921	1.127	596.3	0.042
Chi^2^ (df = 4)	18,461			

## Discussion

Nonoperative management of hemodynamically stable patients with blunt splenic injury has become the standard of care. Literature reports an overall success rate that varies between 78 and 98% [4, 9–13, 16, 17, 20, 28]. In our study nonoperative therapy failed in 11 out of 79 patients (86% success rate) and mortality did not occur.

Patient selection is vital for successful nonoperative management. Only a small group of studies have evaluated the role of concomitant injury. Therefore, our first goal was to determine whether the presence of associated injury affects the outcome of nonoperative treatment. Our second objective was to identify factors that can predict failure. Our study shows that associated injuries are related to prolonged stay on the intensive care and hospitalization, more complications, and increased failure of nonoperative management. The presence of a femur fracture as well as higher age are specific predictors of failure. To our knowledge, this is the first study to show this association.

Previous studies have shown that a higher Injury Severity Score is an independent predictor of failure of conservative therapy [14, 21–23]. Powell et al. [21] found a failure rate of 88% of nonoperatively treated patients with an Injury Severity Score over 15. A multicenter study conducted by the Eastern Association for the Surgery of Trauma that analyzed failure of nonoperative therapy in 917 patients found that an Injury Severity Score > 15 is associated with failure. The failure rate of nonoperative management was 4.0% in patients with an Injury Severity Score < 15 and 13.7% with an Injury Severity Score > 15 (*P* < 0.05) [23].

Moreover, McIntyre et al. found by studying 1633 nonoperatively treated patients that nonoperative therapy was more likely to fail in patients who had an Injury Severity Score > 25 [14].

In our study, univariable analysis confirms a statistical relationship between the need for surgical intervention and a higher Injury Severity Score. However, in our multivariable model analysis no significant relationship between injury severity and failure of nonoperative management was found.

The current analysis shows that the presence of a femur injury increases the chance of failure by a factor of 25.9.

Although the presence of a femur fracture is significantly associated with failure of nonoperative therapy in our multivariable analysis, we do not expect that this injury itself plays a major role in the development of hemodynamic instability resulting in failure of nonoperative management. A detailed evaluation of those patients with a femur fracture showed that none of them had persistent, severe blood loss related to their femoral injury. Therefore, the association of this injury must be interpreted as an expression of high impact trauma.

In the literature, predictors of failure of nonoperative management included higher age [12, 14–16, 29, 30], hemodynamic instability [19, 23, 28], large hemoperitoneum [20, 21], the presence of a contrast blush on CT-scan [17, 18], higher grade of splenic injury, and the presence of associated injuries [14, 21–23].

Hemodynamic instability is considered an absolute contraindication for nonoperative management [19, 23, 28]. All other factors are considered relative contraindications.

The selection criteria for attempting nonoperative treatment remained unchanged during the study period. All hemodynamically stable patients without signs of hollow organ injuries are considered suitable candidates for nonoperative therapy.

In line with the recommendations from Peitzman et al. we utilize strict guidelines for the treatment and monitoring of patients selected for nonoperative therapy [31]. These patients preferably undergo CT-scanning, serial physical examination and measurement of hemodynamic parameters, frequent hemoglobin and hematocrit determinations, bed rest and limited oral intake. Patients with a contrast blush on CT-scan, who become hemodynamically unstable during attempted nonoperative treatment are managed by angio-embolization.

In correlation with previous reports, we found that higher age impaired success of nonoperative management. In the beginning of the conservative therapy-era some studies showed that patients older than 55 years should be treated surgically [14, 15, 29, 30]. Rodrigues et al. found by studying human cadavers that higher age is associated with decreased amounts of elastic fibers in the splenic capsule. This may limit contraction and retraction of damaged vessels in the spleen parenchyma, leading to impaired local hemostasis in the elderly [32]. However, other studies have reported that age does not influence the results of nonoperative therapy [16, 33].

The results of our study demonstrate that higher age is significantly associated with increased likelihood of failure. The median age of our population was 28. The logistic regression analysis showed that the per year increase for the odds of failure is 6%.

There are concerns about the potential for missing hollow viscus organ injuries [34, 35]. Although intra-abdominal hollow organ injury is rare, it carries a high mortality risk [36]. The rate of missed hollow viscus injury in nonoperative management treated blunt splenic injury patients is low, varying between 0 and 1% [19, 20, 25, 28, 34]. Nance et al. showed that in blunt abdominal trauma, the incidence of concurrent hollow viscus injury increases with the number of solid organs injured [37]. In our study, there are no indications that clinically relevant hollow organ injuries were missed. Furthermore, none of the patients who underwent delayed surgical intervention due to failure of nonoperative management had concurrent intra-abdominal hollow organ injuries diagnosed during laparotomy.

Angio-embolization as an adjunct to nonoperative therapy successfully prevented failure in two patients. These two patients became hemodynamically unstable after one day of observation. They were both selected for angio-embolization because active bleeding was found on CT-scan. Following embolization, both patients had an uncomplicated clinical course. This is in line with Brugere et al. who reported very good results of angio-embolization in nonoperatively treated patients with ongoing hemodynamic instability [38]. In our institution, we do not routinely use angio-embolization in hemodynamically stable patients with a contrast blush on CT. Most patients from our study (*n* = 3) with a contrast blush on initial CT were successfully treated without angio-embolization. Univariable analysis shows further that the presence of a contrast blush on initial CT is not associated with failure of nonoperative management. These findings correlate with the study from Olthof et al. in which they found, through a propensity score stratification analysis, that there was no benefit of embolization in patients selected for nonoperative therapy with a contrast blush on CT [39].

A potential confounder of this study is the presence of underlying chronic conditions. However, a chart review of all patients that failed nonoperative management showed that none of these patients suffered underlying chronic disease.

The main strength of the current study is the completeness of patient data. This was achieved by comprehensive documentation of data in a prospectively registered trauma database combined with unlimited access to individual patient records. No patients had to be excluded because of incomplete data.

## Conclusions

We conclude that nonoperative management for blunt splenic injury is safe in *all* hemodynamically normal patients in the absence of concomitant hollow organ injury, even in the presence of severe concurrent injuries or a contrast blush on CT-imaging. These findings are in line with the recommendations from the novel WSES guidelines. In addition we showed that higher age and the presence of a femur fracture are predictive for failure of nonoperative management. In contrast to the literature, a higher Injury Severity Score is not a predictive factor for failure of nonoperative therapy. Furthermore, concomitant injuries are associated with higher failure rates, more complications and increased duration of intensive care unit-stay and overall hospitalization and these patients need to be monitored closely.

## Abbreviations

AIS: Abbreviated Injury Score
APTT: Activated partial thromboplastin time
BPM: Beats per minute
CT: Computed Tomography
GCS: Glasgow Coma Scale
IQR: Inter quartile range
PT: Prothrombin time
SBP: Systolic blood pressure
SPSS: Statistical Package for Social Sciences
WSES: World Society of Emergency Surgery

## References

[1] Davis JJ, Cohn JRI, Nance FC (1976). Diagnosis and management of blunt abdominal trauma. Ann Surg.

[2] Cox EF (1984). Blunt abdominal trauma. A 5-year analysis of 870 patients requiring celiotomy. Ann Surg.

[3] King H, Shumacker HB (1952). Splenic studies. Suspectibility to infection after splenectomy performed in infancy. Ann Surg.

[4] Pachter HL, Guth AA, Hofstetter SR, Spencer FC (1998). Changing patterns in the management of splenic trauma: the impact of nonoperative management. Ann Surg.

[5] Richardson JD (2005). Changes in the management of injuries to the liver and spleen. J Am Coll Surg.

[6] Coccolini F, Montori G, Catena F, Kluger Y, Biffl W, et al. Splenic trauma: WSES classification and guidelines for adult and pediatric patients. WJES. 2017. 10.1186/s13017-017-0151-4. 10.1186/s13017-017-0151-4 PMC5562999 28828034

[7] Rowell SE, Biffl WL, Brasel K, Moore EE, Albracht RA (2017). Western trauma association critical decisions in trauma: the management of adult blunt splenic trauma-2016 updates. J Trauma.

[8] Upadhyaya P, Simpson JS (1969). Splenic trauma in children. Surg Gynecol Obstet.

[9] Dent D, Alsabrook G, Erickson BA, Meyers J, Wholey M (2004). Blunt splenic injuries: high nonoperative management rate can be achieved with selective embolization. J Trauma.

[10] Hunt JP, Lentz CW, Cairns BA, Ramadan FM, Smith DL (1996). Management and outcome of splenic injury: the results of a five-year statewide population-based study. Am Surg.

[11] Giannopoulos George A, Katsoulis Iraklis E, Tzanakis Nikolaos E, Patsaouras Panayotis A, Digalakis Michalis K (2009). Non-operative management of blunt abdominal trauma. Is it safe and feasible in a district general hospital?. Scandinavian Journal of Trauma, Resuscitation and Emergency Medicine.

[12] Harbrecht BG, Peitzman AB, Rivera L, Heil B, Croce M (2001). Contribution of age and gender to outcome of blunt splenic injury in adults: multicenter study of the eastern association for the surgery of trauma. J Trauma.

[13] Myers JG, Dent DL, Stewart RM, Gray GA, Smith DS (2000). Blunt splenic injuries: dedicated trauma surgeons can achieve a high rate of nonoperative success in patients of all ages. J Trauma.

[14] McIntyre LK, Schiff M, Jurkovich GJ (2005). Failure of nonoperative management of splenic injuries: causes and consequences. Arch Surg.

[15] Godley CD, Warren RL, Sheridan RL, McCabe CJ (1996). Nonoperative management of blunt splenic injury in adults: age over 55 as a powerful indicator for failure. J Am Coll Surg.

[16] Cocanour CS, Moore FA, Ware DN, Marvin RG, Duke JH (2000). Age should not be a consideration for nonoperative management of blunt splenic injury. J Trauma.

[17] Schurr MJ, Fabian TC, Gavant M, Croce MA, Kudsk KA (1995). Management of blunt splenic trauma: computed tomographic contrast blush predicts failure of nonoperative management. J Trauma.

[18] Omert LA, Salyer D, Dunham CM, Porter J, Silva A (2000). Implications of the “contrast blush” finding on computed tomogaphic scan of the spleen in trauma. J Trauma.

[19] Haan JM, Bochicchio GV, Kramer N, Scalea TM (2005). Nonoperative management of blunt splenic injury: a 5-year experience. J Trauma.

[20] Velhamos GC, Toutouzas KG, Radin R, Chan L, Demetriades D (2003). Nonoperative treatment of blunt injury to solid abdominal organs: a prospective study. Arch Surg.

[21] Powell M, Courcoulas A, Gardner M, Lynch J, Harbrecht BG (1997). Management of blunt splenic trauma: significant differences between adults and children. Surgery.

[22] Malhotra AK, Latifi R, Fabian TC, Ivatury RR, Dhage S (2003). Multiplicity of solid organ injury: influence on management and outcomes after blunt abdominal trauma. J Trauma.

[23] Peitzman AB, Heil B, Rivera L, Federle MB, Harbrecht BG (2000). Blunt splenic injury in adults: multi-institutional study of the eastern Association for the Surgery of trauma. J Trauma.

[24] Yanar H, Ertekin C, Taviloglu K, Kabay B, Bakkaloglu H (2008). Nonoperative treatment of multiple intra-abdominal solid organ injury after blunt abdominal trauma. J Trauma.

[25] Sartorelli KH, Frumiento C, Rogers FB, Osler TM (2000). Nonoperative management of hepatic, splenic, and renal injuries in adults with multiple injuries. J Trauma.

[26] MacLeod JB, Lynn M, McKenney MG, Cohn SM, Murtha M (2003). Early coagulopathy predicts mortality in trauma. J Trauma.

[27] Greenspan L, McLellan BA, Greig HRN (1985). Abbreviated injury scale and injury severity score: a scoring chart. J Trauma.

[28] Cogbill TH, Moore EE, Jurkovich GJ, Mooris JA, Mucha P (1989). Nonoperative mangemenent of blunt splenic trauma: a multicenter experience. J Trauma.

[29] Renzulli P, Gross T, Schnüringer B, Schoepfer AM, Inderbitzin D (2010). Management of blunt injuries to the spleen. Br J Surg.

[30] Ong AW, Eilertson KE, Reilly EF, Geng TA, Madbak F (2016). Nonoperativ management of splenic injuries: significance of age. J Surg Res.

[31] Peitzman AB, Harbrecht BG, Rivera L, Heil B (2005). EAST multiinstitutional trails workgroup. Failure of observation of blunt splenic injury in adults: variability in practice and adverse consequences. J Am Coll Surg.

[32] Rodrigues CJ, Sacchetti JC, Rodrigues AJ (1999). Age-related changes in the elastic fiber network of the human splenic capsule. Lymphology.

[33] Barone JE, Burns G, Svehlak SA, Tucker JB, Bell T (1999). Management of blunt splenic trauma in patients older than 55 years. Southern Connecticut regional trauma quality assurance committee. J Trauma.

[34] Miller PR, Croce MA, Bee TK, Malhotra AK, Fabian TC (2002). Associated injuries in blunt solid organ trauma: implications for missed injury in nonoperative management. J Trauma.

[35] Kemmeter PR, Hoedema RE, Foote JA, Scholten DJ (2001). Concomitant blunt enteric injuries with injuries of the liver and spleen: a dilemma for trauma surgeons. Am Surg.

[36] Watts DD, Fakhry SM (2003). EAST multi-institutional hollow viscus injury research group. Incidence of hollow viscus injury in blunt trauma: an analysis from 275,557 trauma admissions from the East multi-institutional trial. J Trauma.

[37] Nance ML, Peden GW, Shapiro MB, Kauder DR, Rotondo MF (1997). Solid viscus injury predics major hollow viscus injury in blunt abdominal trauma. J Trauma.

[38] Brugere C, Arvieux C, Dubuisson V, Dubuisson V, Guillon F (2008). Early embolization in the nonoperative management of blunt splenic injuries: a retrospective multicenter study. J Chir (Paris).

[39] Olthof DC, Joose P, Bossuyt PM, de Rooij PP, Leenen LP (2016). Observation versus embolization in patients with blunt splenic injury after trauma: a prospensity score analysis. World J Surg.

